# Microbial community structure of cigar tobacco leaves in Hunan production area and its regulatory mechanism on quality formation

**DOI:** 10.3389/fmicb.2026.1959028

**Published:** 2026-10-28

**Authors:** Wen Cai, Qianying Zhang, Jie Liu, Wanrong Hu, Zhishun Chai, Yonghao Tan, Lei Xing, Dongliang Li

**Affiliations:** 1 China Tobacco Sichuan Industrial Co., Ltd., Chengdu, China; 2 School of Bioengineering, Chongqing University, Chongqing, China

**Keywords:** cigar tobacco leaves, flavor characteristic, high-throughput sequencing, microbial community, origin difference analysis, sensory evaluation

## Abstract

**Introduction:**

To elucidate the microbial community structure of domestic cigar tobacco leaves and its regulatory function in quality formation, the present work focuses on characterizing microbial flora, identifying origin-mediated community divergence, and exploring their linkages to flavor quality for cigar tobacco planted in Hunan.

**Methods:**

High-throughput sequencing, chemical profiling and sensory evaluation were combined to systematically analyze different cigar varieties from the Hunan production region. ANOSIM and correlation network analysis were performed to evaluate microbial community variation and inter-taxa correlations.

**Results and discussion:**

Bacterial communities were dominated by *Proteobacteria*, *Cyanobacteria*, *Firmicutes*, and *Actinobacteria*; fungi were overwhelmingly dominated by *Ascomycota* (relative abundance >93%). At genus level, dominant bacteria included *Pantoea*, *Pseudomonas*, *Klebsiella*, *Brachybacterium*, and *Curtobacterium*, while dominant fungi were *Aspergillus*, *Gibberella*, and *Alternaria*. ANOSIM (R = 0.50, *P* = 0.001) demonstrated that geographical origin acted as the primary driver of microbial community differentiation, and the same cultivar Cuba No.7 showed highly significant interregional differences (R = 0.95, *P* = 0.001). Correlation network showed flavor-positive genera (*Pantoea*, *Curtobacterium*, *Aureimonas*) were enriched in Yongding District samples, whereas flavor-negative genus *Trichoderma* was abundant in Sangzhi County and Chenzhou samples. Unsaturated fatty acids, succinic acid and malic acid were key metabolites connecting microbial communities and sensory traits. These results support the hypothesis that microorganisms dominate quality formation of cigar tobacco leaves, and offer theoretical support for site-specific cultivation and fermentation optimization based on regional microecological features.

## 1 Introduction

Cigars are a premium tobacco product made from cured and fermented leaves (Jin, 1988). In recent years, the rapid growth of cigar sales in the Chinese market has underscored the industry’s considerable potential and driven a steady rise in demand for raw materials (Zhou et al., 2021). The quality of cigar tobacco leaves is therefore a key determinant for the sustainable development of the sector. Hunan Province, one of China’s major tobacco-growing regions, began pilot cultivation of cigar tobacco in 2021. Several studies have since addressed this emerging crop: Wang classified Hunan into potential zones for wrapper production and for combined filler-and-wrapper cultivation, based on climatic conditions, using correlation analysis between leaf chemical components and environmental factors (Wang et al., 2023). Guo et al. (2023) compared the post-fermentation quality traits of different cigar varieties grown in the hilly and mountainous areas of the province. Xu et al. (2023) evaluated field growth performance, disease incidence, economic traits, and quality indicators across various cigar varieties in Hunan, assessing their regional suitability. Given this background, research on the production techniques and underlying mechanisms of these trial varieties across Hunan’s production areas is essential for enhancing their industrial usability and overall commercial value.

Cigar tobacco leaves from different growing regions are known to develop distinct flavor profiles, making systematic evaluation of their sensory characteristics essential for blending compatibility studies (Li et al., 2019; Wang et al., 2020). Meanwhile, microorganisms play a pivotal role in the fermentation of cigar tobacco leaves (Zhang Q. Y. et al., 2020), and regional differences may give rise to unique microbial communities that influence final quality. Understanding the composition and function of these communities can provide valuable guidance for process optimization (Zhang Q. Y. et al., 2020). During fermentation, the indigenous microbial communities on the surface of tobacco leaves utilize the organic compounds in the leaves as substrates, degrading and converting macromolecular substances such as proteins, starch, lignin, and nicotine into amino acids, alcohols, aldehydes, ketones, organic acids, and other heterocyclic aromatic compounds, which either directly constitute aroma components or serve as flavor precursors (Yue et al., 2026). Specifically, the core bacterial communities dominated by *Pseudomonas*, *Staphylococcus*, and *Cyanobacteria*, together with metabolically active fungi such as *Aspergillus*, *Penicillium*, and *Inocybe*, have been identified as key drivers of carbon–nitrogen coupled metabolism during fermentation. Functional strains including *Pseudomonas* fulva, *Staphylococcus* nepalensis, *Bacillus subtilis*, *Stenotrophomonas rhizophila*, *Alternaria alternata*, and *Aspergillus cristatus* have been demonstrated to actively degrade carbon- and nitrogen-containing compounds, thereby promoting the release of volatile flavor compounds. Moreover, cross-kingdom interactions between bacterial and fungal communities—typically exemplified by the synergistic cooperation between *Pseudomonas* and *Aspergillus* as functional hubs—collectively maintain the stability of the fermentation ecosystem and may participate in nicotine degradation and the formation of volatile compounds (Zhang et al., 2025c). Beyond degradation, microorganisms also participate in the synthesis and degradation metabolism of various amino acids, alkaloids, and flavonoids, which constitute key metabolic pathways for the formation of characteristic flavor in cigar tobacco leaves. These microbial activities ultimately lead to a reduction in irritating compounds, an enhancement of aroma complexity, and an overall improvement in tobacco leaf quality (Zhang Y. et al., 2026). Recent advances in high-throughput sequencing have enabled the application of multiple omics approaches—including genomics, transcriptomics, proteomics, and metabolomics—to investigate cigar curing and fermentation (Song et al., 2017; Yan et al., 2022).

In this study, we integrate microbial high-throughput sequencing, bioinformatics, and metabolomic profiling to identify core microorganisms associated with cigar tobacco leaves and to elucidate their contributions to flavor quality, with the ultimate goal of improving the quality of domestically produced cigar tobacco leaves. Specifically, we systematically evaluate the flavor characteristics of post-agricultural-fermentation leaves from various production areas in Hunan Province, and comprehensively analyze their microbial community structures. This work aims to provide theoretical support for quality improvement and product application innovation in Hunan’s cigar tobacco industry.

## 2 Materials and methods

### 2.1 Materials

A total of 11 samples were included in this study, covering six cigar varieties cultivated across five production areas in Hunan Province. All samples were middle leaves that had undergone agricultural fermentation, with three biological replicates collected per sample. Detailed sample information is provided in Table 1. In this context, agricultural fermentation is defined as the initial post-harvest processing step in which air-cured cigar tobacco leaves are stacked to undergo a preliminary fermentation. During this stage, endogenous enzymes and epiphytic microorganisms act synergistically to degrade macromolecular substrates and promote the accumulation of aroma precursors, thus constituting a fundamental stage in the quality formation of cigar raw materials.

**TABLE 1:** Sample information.

Sample	Production areas	Variety	Crop year	Leaf position
TL-1	Yongding District, Zhangjiajie	Yunxue No. 2	2022	Middle
TL-2	Yongding District, Zhangjiajie	Cuba No. 7	2022	Middle
TL 3	Yongding District, Zhangjiajie	Habanos	2022	Middle
TL-4	Cili County, Zhangjiajie	Cuba No.7	2022	Middle
TL-5	Cili County, Zhangjiajie	CX081	2022	Middle
TL-6	Sangzhi County, Zhangjiajie	Cuba No.7	2022	Middle
TL-7	Sangzhi County, Zhangjiajie	Habanos	2022	Middle
TL-8	Yongzhou	Yunxue No. 2	2022	Middle
TL-9	Yongzhou	Cuba No. 7	2022	Middle
TL-10	Chenzhou	ZX251	2022	Middle
TL-11	Chenzhou	ZX158	2022	Middle

### 2.2 Agricultural fermentation conditions

The cigar tobacco leaves were conditioned using two methods: layer-by-layer spray humidification and steam humidification on hanging rods. The moisture content of the middle leaves was adjusted to 27% ± 2%. For fermentation, cigar tobacco leaves were typically stacked in piles. Each pile contained approximately 2,000 kg of tobacco leaves, with a height of 1.5–1.8 m, a width of 2.0–2.4 m, and a length starting from 3.6 m, which was extended depending on the total leaf quantity. The pallets used had dimensions of 1.0 m × 1.2 m.

During fermentation, the temperature inside each pile was monitored twice daily. When the core temperature reached the preset maximum turning temperature of 45 °C, the pile was promptly turned. During turning, each bundle of leaves was shaken and loosened to dissipate heat and remove off-odors. In addition, if the core temperature failed to rise to the preset maximum temperature and remained stable or slightly decreased for 2–3 consecutive days, the pile was also turned in a timely manner. At that point, smaller piles were combined into larger ones and restacked to continue fermentation.

The fermentation process was terminated when the technical evaluators determined that the sensory smoking quality, appearance quality, and smoldering duration of the leaves had all met the predetermined targets. Throughout the fermentation period, the ambient temperature in the fermentation room was maintained at no less than 25 °C, and the relative humidity was kept between 70% and 90%.

### 2.3 Sensory evaluation

The tobacco leaves were hand-rolled into cigars of 38 ring gauge and 110 mm length. The cigars were conditioned in a temperature- and humidity-controlled chamber at 20 °C and 65 % relative humidity for 30 days to equilibrate moisture content. Seven trained panelists assessed the samples according to the sensory evaluation criteria outlined in Table 2. Sensory evaluation covered three main categories: aroma note characteristics, off-notes, and overall quality attributes.

**TABLE 2:** Evaluation table of sensory quality and style of domestic cigar tobacco leaves.

Sample	TL-1	TL-2	TL-3	TL-4	TL-5	TL-6	TL-7	TL-8	TL-9	TL-10	TL-11
Nutty	0	1	0	0	0	0	0	0	0	0	0
Bean	0	0	0	0	0	0	0	0	1	0	0
Woody	0	0	1	0	0	1	1	0	1	1	2
Fruity	0	0	0	0	0	0	0	0	0	0	0
Fresh sweet	0	1	1	0	3	1	3	0	0	0	0
Caramel sweet	0	3	0	0	0	0	0	0	0	3	3
Honey sweet	3	0	3	3	0	3	0	3	3	0	0
Floral	2	0	1	1	0	1	0	1	0	0	0
Creamy	0	1	0	0	0	0	0	0	0	0	0
Roasted	2	1	1	1	1	1	1	2	2	2	1
Hay	1	1	1	1	1	1	1	0	0	1	1
Powdery	2	0	0	2	2	0	0	1	0	0	0
Protein off-note	0	0	0	0	0	0	0	1	1	0	0
Earthy off-note	0	0	0	0	0	0	0	0	0	0	0
Green harsh off-note	1	0	0	1	1	0	0	1	1	1	0
Total aroma volume	6.5	6.5	6.5	6.5	6	6.5	6.5	6	6	6	6
Aroma richness	7	7	6	6.5	6	6	6.5	6	6.5	6	6.5
Maturity	6.5	6.5	6.5	6.5	6	6.5	6.5	6.5	6.5	6	6
Irritation	6.5	6.5	6.5	6.5	6	6.5	6.5	6.5	6.5	6	6
Smoothness	6.5	6.5	6	6.5	6	6	6	6	6	6	6
Fineness	6	6.5	6	6	6	6	6	6	6	6.5	6
Sweetness and moistness	6	6.5	6	6	6	6	6.5	6.5	6	6.5	6
Cleanliness	6	6.5	6.5	6.5	6	6.5	6.5	6	6	6	7
Aftertaste	6	6.5	6.5	6	6	6.5	6.5	6	6	5.5	6.5
Combustibility	6	6.5	6.5	6.5	6.5	6.5	6	6.5	6.5	6.5	6
Ash color	6	6	6	6	6	6	6	6.5	6.5	6	5.5
Ash cohesion	7	6	6	7	6	6	6	6.5	6.5	6	5.5

### 2.4 Determination of microbial diversity

Total genomic DNA was extracted from each sample using the E.Z.N.A.^®^ Soil DNA Kit (Omega Bio-tek, Norcross, GA, USA) following the manufacturer’s instructions. DNA quality was assessed by 1% agarose gel electrophoresis, and DNA concentration and purity were measured with a NanoDrop 2000 spectrophotometer. The bacterial 16S rRNA gene V3–V5 region was amplified using universal primers, while the fungal internal transcribed spacer (ITS1) region was amplified with primers ITS1 and ITS4. PCR products were excised from 2% agarose gels, purified with the AxyPrep DNA Gel Extraction Kit (Axygen Biosciences, Union City, CA, USA), and quantified using a Quantusï™ Fluorometer. Sequencing libraries were prepared with the NEXTFLEX Rapid DNA-Seq Kit (New England Biolabs Inc., Ipswich, MA, USA) and sequenced on an Illumina MiSeq PE300 platform (Illumina Corporation, San Diego, USA). Raw reads were assembled using FLASH, and operational taxonomic units (OTUs) were clustered and chimeras removed using USEARCH v7.0. Rarefaction curves, taxonomic composition, and abundance distribution tables were subsequently generated.

### 2.5 Determination of petroleum ether extract content

The petroleum ether extract content in tobacco leaf samples was determined using a Soxhlet extractor. Accurately weighed ground tobacco leaf samples (3.0 g) were soaked in petroleum ether at room temperature for 0.5 h, followed by extraction at 65 °C for 2.0 h, rinsing for 0.5 h, and evaporation of the solvent for 0.5 h. The samples were then placed in an oven and dried to constant weight. The petroleum ether extract content was subsequently obtained by gravimetric analysis. Petroleum ether extract content was determined following the method described by Li et al. (2022).

### 2.6 Determination of non-volatile organic acid content

The non-volatile organic acids content in tobacco leaf samples was determined by methyl esterification coupled with gas chromatography. The methyl-esterified samples were injected into a gas chromatograph. The chromatographic conditions were as follows: a Thermo Fisher Trace TR-FAME column (60 m × 0.25 mm × 0.25 μm); the temperature program was held at 130 °C for 3 min, then increased to 200 °C at 5 °C/min and held for 10 min, followed by an increase to 220 °C at 2 °C/min and held for 3 min; the column flow rate was 1.8 mL/min. Non-volatile organic acids were analyzed following the method described by Fu et al. (2024).

### 2.7 Data processing

Differences among sample data were analyzed using SPSS 26.0 statistical software. Microbial community analysis was performed on the Majorbio Cloud Platform.^1^ Experimental data were organized using Excel. The Spearman correlation coefficients between fungal/bacterial communities and chemical composition indices as well as sensory evaluation indices were systematically analyzed using R software, and correlation network diagrams were generated. Except for the microbial alpha diversity analysis, which is presented as mean values alone, all other numerical data in the figures and tables throughout the manuscript are expressed as mean ± standard deviation (SD). Given the limited sample size, no inferential statistical analysis was performed, and the results are primarily presented as descriptive comparisons.

## 3 Results

### 3.1 Sensory quality evaluation

As shown in Figure 1A, marked differences in characteristic aroma notes were observed among samples from different growing regions and varieties. The predominant aroma profiles were primarily fresh sweet, caramel sweet, and honey sweet notes, with some samples also exhibiting secondary notes such as roasted, powdery, woody, beany, and nutty aromas.

**FIGURE 1:**
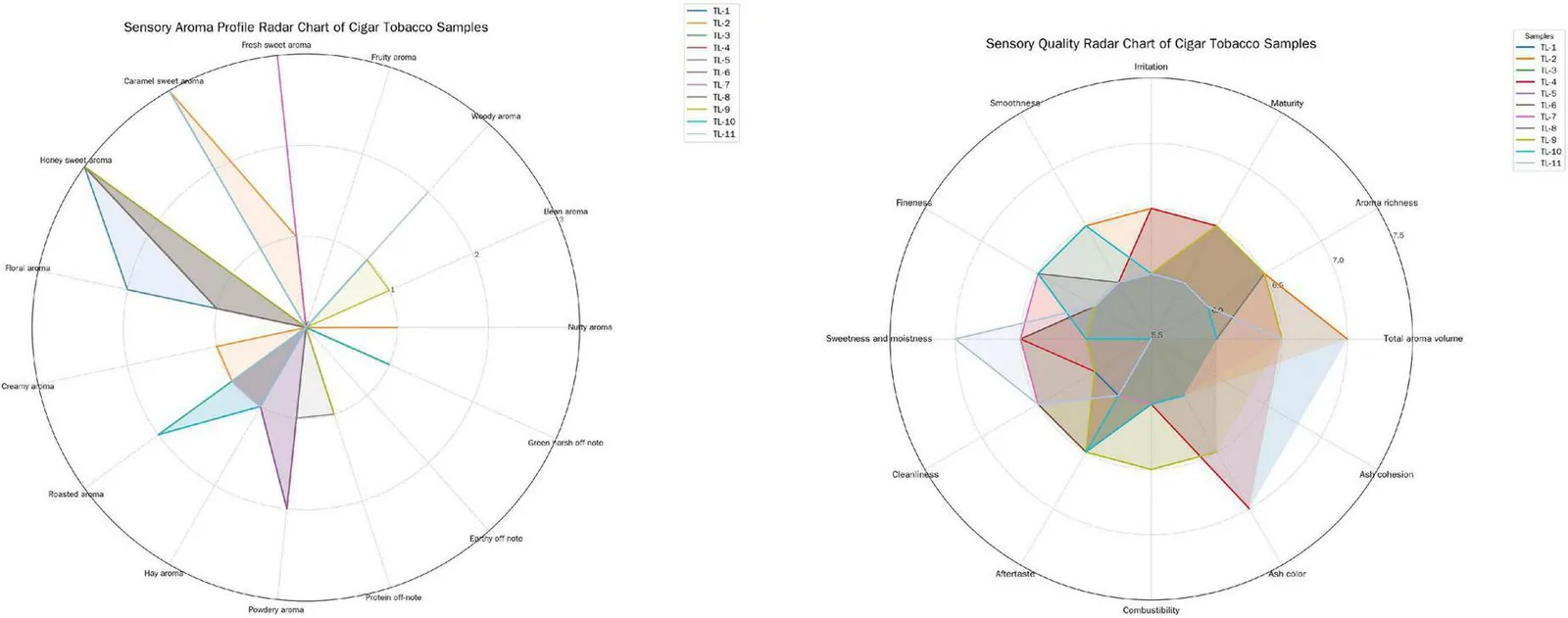
Evaluation scores of cigar tobacco samples.

More specifically, samples of cultivar CX081 from Cili County (TL-5) and Habanos from Sangzhi County (TL-7) were characterized by a dominant fresh sweet note. Cuba No.7 from Yongding District (TL-2), as well as ZX158 (TL-11) and ZX251 (TL-10) from Chenzhou City, featured caramel sweet as their core aroma. Honey sweet notes prevailed in Yunxue No.2 (TL-1) and Habanos from Yongding District (TL-3), Cuba No.7 from both Cili (TL-4) and Sangzhi Counties (TL-6), and Yunxue No.2 (TL-8) and Cuba No.7 (TL-9) cultivar from Yongzhou City.

The overall sensory profiles of the tobacco leaves are summarized in Figure 1B. Samples from Yongding District and Sangzhi County scored highest in overall aroma and smoke characteristics. Leaves from Cili County performed particularly well in smoke and aftertaste. Those from Yongzhou City outperformed the other regions in aroma quality and combustibility, whereas samples from Chenzhou City exhibited the most balanced performance across all sensory indicators. As shown in Table 3, samples from Yongding District exhibited relatively higher mean scores in key attributes, including total aroma volume, aroma richness, and maturity, whereas those from Cili County performed better in combustibility and ash cohesion. In contrast, Chenzhou samples received comparatively lower scores in ash color and ash cohesion. Notably, Yongding District had the highest richness score among all production areas. According to Table 4, Cuba No. 7 had the largest sample size among all varieties and demonstrated stable performance across multiple indicators. Yunxue No. 2 showed better scores in maturity, irritation, and ash cohesion, while Habanos ranked intermediate in most attributes. Varieties with insufficient sample sizes were not subjected to reliable statistical comparisons.

**TABLE 3:** Statistical analysis of quality characteristic scores for each producing area.

Quality characteristics	Yongding	Cili	Sangzhi	Yongzhou	Chenzhou
Aroma quantity	6.50 ± 0.00	6.25 ± 0.35	6.50 ± 0.00	6.00 ± 0.00	6.00 ± 0.00
Richness	6.67 ± 0.58	6.25 ± 0.35	6.25 ± 0.35	6.25 ± 0.35	6.25 ± 0.35
Maturity	6.50 ± 0.00	6.25 ± 0.35	6.50 ± 0.00	6.50 ± 0.00	6.00 ± 0.00
Harshness	6.50 ± 0.00	6.25 ± 0.35	6.50 ± 0.00	6.50 ± 0.00	6.00 ± 0.00
Smoothness	6.33 ± 0.29	6.25 ± 0.35	6.00 ± 0.00	6.00 ± 0.00	6.00 ± 0.00
Delicacy	6.17 ± 0.29	6.00 ± 0.00	6.00 ± 0.00	6.00 ± 0.00	6.25 ± 0.35
Sweetness	6.17 ± 0.29	6.00 ± 0.00	6.25 ± 0.35	6.25 ± 0.35	6.25 ± 0.35
Cleanliness	6.33 ± 0.29	6.25 ± 0.35	6.25 ± 0.35	6.00 ± 0.00	6.50 ± 0.71
Aftertaste	6.33 ± 0.29	6.00 ± 0.00	6.50 ± 0.00	6.00 ± 0.00	6.00 ± 0.71
Combustibility	6.33 ± 0.29	6.50 ± 0.00	6.25 ± 0.35	6.50 ± 0.00	6.25 ± 0.35
Ash color	6.00 ± 0.00	6.00 ± 0.00	6.00 ± 0.00	6.50 ± 0.00	5.75 ± 0.3
Ash cohesiveness	6.33 ± 0.58	6.50 ± 0.71	6.00 ± 0.00	6.50 ± 0.00	5.75 ± 0.35

**TABLE 4:** Statistical analysis of quality characteristic scores for each variety.

Quality characteristics	Cuba No. 7	Yunxue No. 2	Habanos	CX081	ZX251
Total aroma volume	6.38 ± 0.25	6.33 ± 0.29	6.25 ± 0.35	6.00	6.00
Richness	6.50 ± 0.41	6.33 ± 0.58	6.25 ± 0.35	6.00	6.00
Maturity	6.38 ± 0.25	6.50 ± 0.00	6.50 ± 0.00	6.00	6.00
Irritation	6.38 ± 0.25	6.50 ± 0.00	6.50 ± 0.00	6.00	6.00
Smoothness	6.25 ± 0.29	6.17 ± 0.29	6.00 ± 0.00	6.00	6.00
Finenss	6.00 ± 0.00	6.17 ± 0.29	6.00 ± 0.00	6.00	6.50
Sweetness and moistness	6.25 ± 0.29	6.17 ± 0.29	6.25 ± 0.35	6.00	6.50
Cleanliness	6.38 ± 0.25	6.17 ± 0.29	6.25 ± 0.35	6.00	6.00
Aftertaste	6.25 ± 0.29	6.17 ± 0.29	6.25 ± 0.35	6.00	5.50
Combustibility	6.50 ± 0.00	6.33 ± 0.29	6.25 ± 0.35	6.50	6.50
Ash color	6.13 ± 0.25	6.17 ± 0.29	6.00 ± 0.00	6.00	6.00
Ash cohesion	6.13 ± 0.25	6.33 ± 0.58	6.00 ± 0.00	6.50	6.00

### 3.2 Microbial diversity

Amplicon sequencing targeting the bacterial 16S rRNA gene and the fungal ITS region was performed on tobacco leaf samples using the Illumina MiSeq platform. After quality filtering and chimera removal, we obtained 2,565,656 high-quality bacterial reads and 2,679,801 fungal reads. Alpha diversity indices of the microbial communities are summarized in Table 5. All samples exhibited coverage values exceeding 0.999, indicating sufficient sequencing depth to comprehensively capture the true community structure of the microorganisms associated with the tobacco leaves.

**TABLE 5:** Alpha diversity of microbes in cigar leaves.

Sample	Bacteria			Fungi		
	Chao1	Shannon	Coverage	Chao1	Shannon	Coverage
TL-1	249.03	1.71	0.999	140.40	1.51	0.999
TL-2	434.00	2.55	0.999	48.88	0.74	1.000
TL-3	259.58	1.66	0.999	127.94	1.09	0.999
TL-4	425.82	2.80	0.999	162.68	1.81	0.999
TL-5	270.49	2.32	0.999	297.96	2.75	0.998
TL-6	180.25	0.69	0.999	240.14	2.49	0.998
TL-7	285.00	1.77	0.999	110.67	1.40	0.999
TL-8	334.83	2.81	0.999	224.50	2.05	0.999
TL-9	225.60	1.05	0.999	173.73	2.34	0.999
TL-10	168.00	1.55	0.999	174.12	2.41	0.999
TL-11	137.67	1.12	1.000	189.67	2.03	0.999

The Chao1 index was used to estimate microbial community richness, while the Shannon index served as a measure of community diversity. As shown in Table 3, Chao1 values ranged from 137.67 to 434.00 for bacteria and from 48.88 to 297.96 for fungi, indicating that bacterial richness was substantially higher than fungal richness across all samples.

Notable differences in both richness and diversity were observed among production areas for the same cultivar. For Yunxue No. 2, both bacterial and fungal diversity followed the order: Yongzhou (TL-9) > Yongding (TL-2) District. For Cuba No. 7, bacterial richness and diversity were highest in Yongding District (TL-2) and lowest in Sangzhi County (TL-6); conversely, fungal diversity was highest in Sangzhi County (TL-6) and lowest in Yongding District (TL-2), showing a reverse pattern. Similarly, the Habanos variety exhibited regional variation: samples from Sangzhi County (TL-7) displayed the highest bacterial richness and diversity but the lowest fungal diversity, whereas samples from Yongding District (TL-3) showed the opposite trend.

### 3.3 Analysis of microbial community structure

#### 3.3.1 Analysis of microbial community composition

The phylum-level community compositions are presented in Figures 2A,B. The predominant bacterial phyla were *Proteobacteria*, *Cyanobacteria*, *Firmicutes*, *Actinobacteria*, along with several unclassified phyla. For fungi, the dominant phyla were *Ascomycota*, *Basidiomycota*, and *Mortierellomycota*, together with unclassified groups.

**FIGURE 2:**
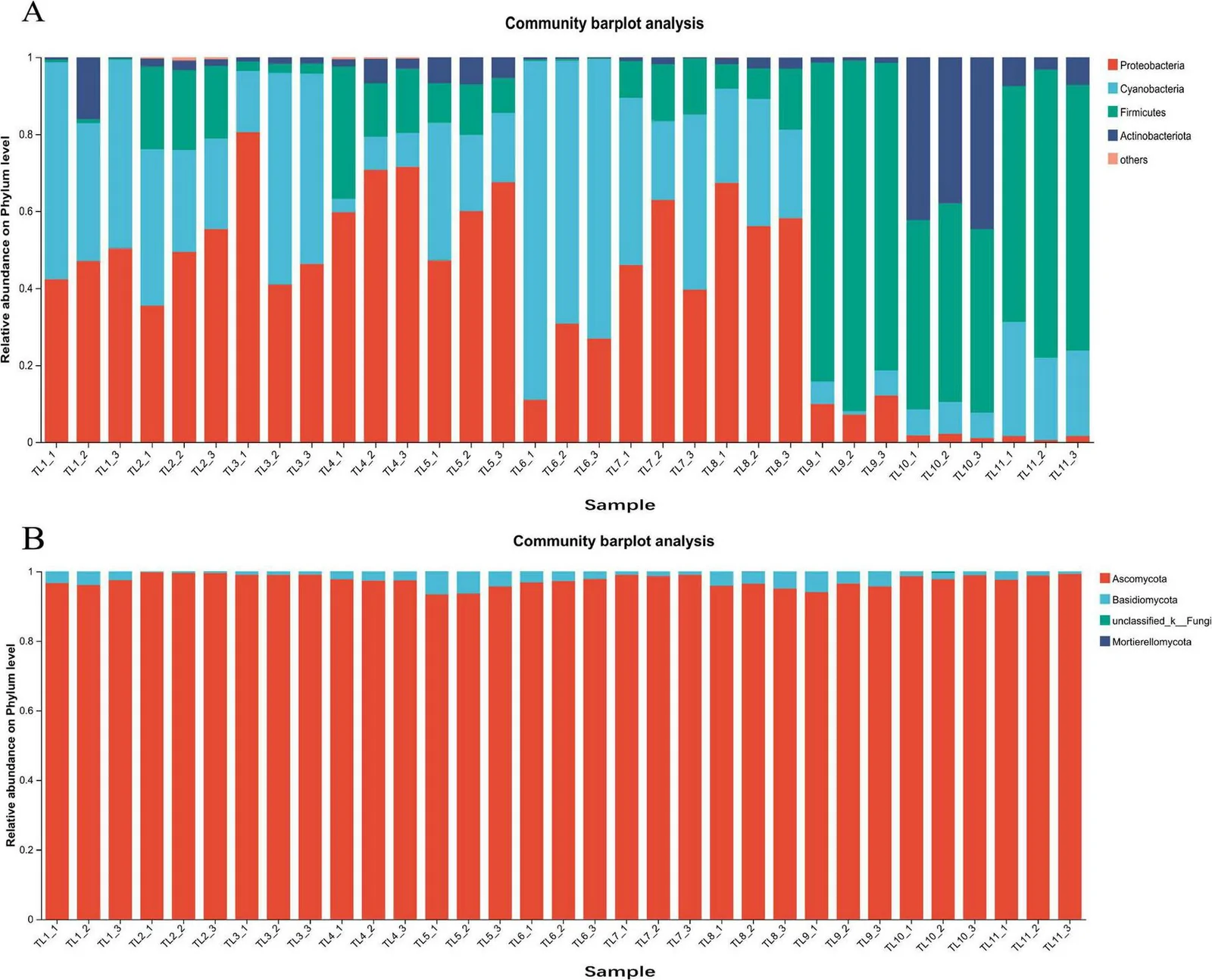
Plot of phylum level relative abundances of bacterial and fungal communities in cigar leaves. **(A)** Bacterial community composition at the phylum level. **(B)** Fungal community composition at the phylum level. The x-axis represents individual samples (TL-1 to TL-11), and the y-axis indicates the relative abundance (0–1). Each bar corresponds to one sample, with different colors representing different phyla. Taxa with relative abundance below 1% were grouped as “Others.” The dominant bacterial phyla were *Proteobacteria*, *Cyanobacteria*, *Firmicutes*, and *Actinobacteria*, while *Ascomycota* was the overwhelmingly dominant fungal phylum across all samples (relative abundance >93%). Data were derived from high-throughput sequencing of the 16S rRNA gene (bacteria) and the ITS region (fungi).

The dominant phyla varied considerably across production areas and cultivars. In the three Zhangjiajie production areas, *Proteobacteria* was the dominant bacterial phylum for most samples, with relative abundances ranging from 35.89% to 80.35%, except for Cuba No. 7, where *Cyanobacteria* dominated (68.33%–88.11%). In Yongzhou, *Proteobacteria* was dominant for Yunxue No. 2(TL-8) (56.11%–67.34%), whereas *Firmicutes* dominated for Cuba No. 7(TL-8) (79.94%–91.02%). In Chenzhou, *Firmicutes* was the dominant phylum across all samples (61.28%–74.86%). In contrast, *Ascomycota* was overwhelmingly the dominant fungal phylum across all samples, with relative abundances ranging from 93.43% to 99.80%.

The genus-level community profiles are shown in Figures 3A,B. The dominant bacterial genera included *Pantoea*, *Pseudomonas*, *Klebsiella*, *Aerococcus*, *Bacillus*, *norank*_*f*_*Mitochondria*, *Corynebacterium*, *Enterobacter*, *Brachybacterium*, *Acinetobacter*, *Curtobacterium*, and *Pectobacterium*. The dominant fungal genera comprised *Aspergillus*, *Alternaria*, *Gibberella*, *Sampaiozyma*, *Cercospora*, and *Corynespora*.

**FIGURE 3:**
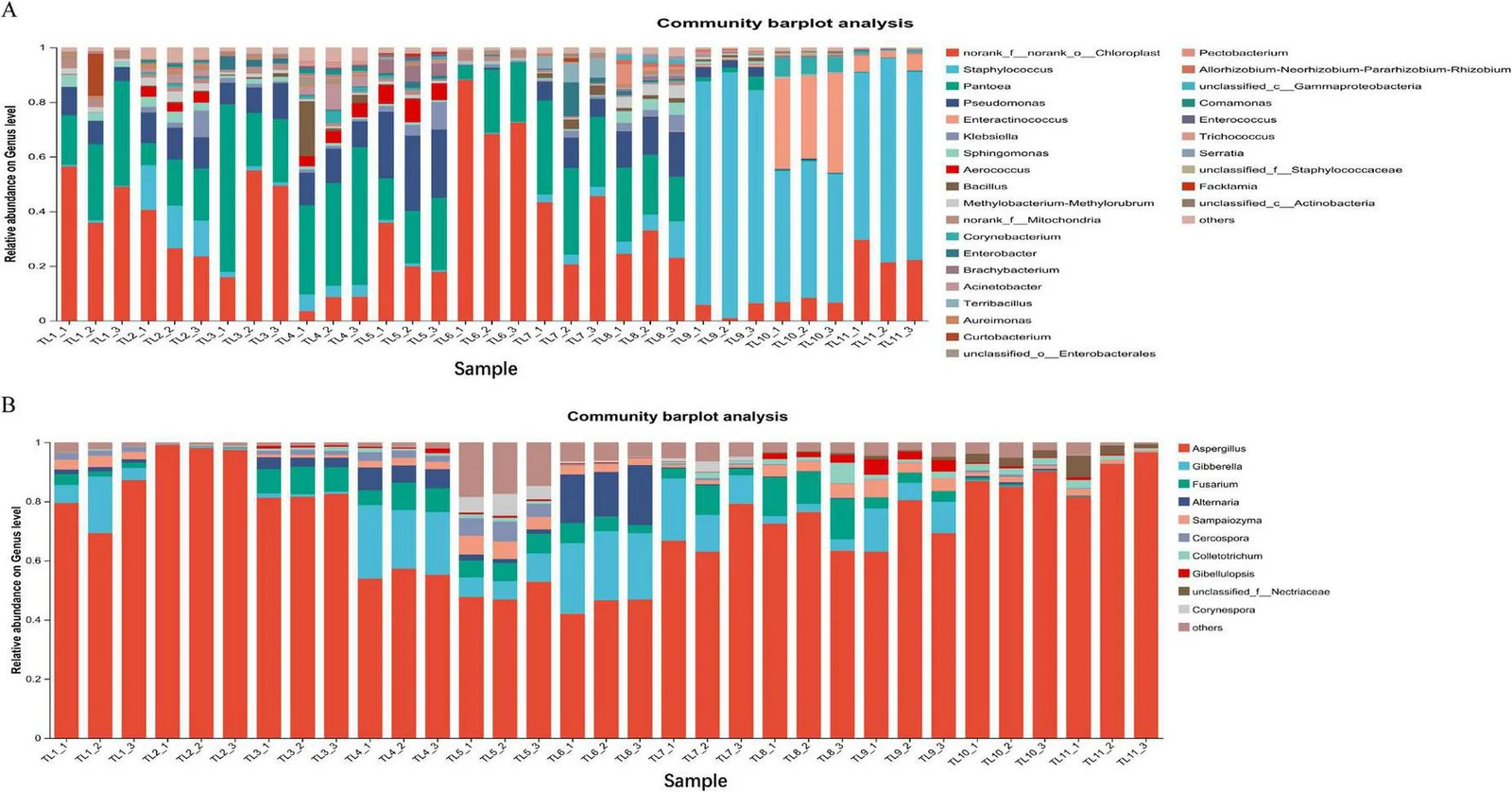
Plot of genus level relative abundances of bacterial and fungal communities in cigar leaves. **(A)** Bacterial community composition at the genus level. **(B)** Fungal community composition at the genus level. The x-axis represents individual samples (TL-1 to TL-11), and the y-axis indicates the relative abundance (0–1). Different colors denote different genera. The top 20 most abundant genera are shown, and the remaining genera are collectively labeled as “Others.” The dominant bacterial genera included *Pantoea*, *Pseudomonas*, *Klebsiella*, *Aerococcus*, *Bacillus*, *Corynebacterium*, *Enterobacter*, *Brachybacterium*, *Acinetobacter*, *Curtobacterium*, and *Pectobacterium*; the dominant fungal genera included *Aspergillus*, *Alternaria*, *Gibberella*, *Sampaiozyma*, *Cercospora*, and *Corynespora*. Data were based on 16S rRNA and ITS amplicon sequencing.

At the bacterial genus level, the community composition of Cuba No. 7 in Sangzhi County (TL-6) differed markedly from that in Yongding District (TL-2) and Cili County (TL-4): *Pseudomonas* was relatively abundant in Yongding and Cili (9.43%–12.67%), but its relative abundance in Sangzhi was only about 0.34%. At the fungal genus level, the composition of Cuba No. 7 in Yongding District (TL-2) differed sharply from that in Sangzhi (TL-6) and Cili (TL-4): *Gibberella* was prominent in Sangzhi and Cili (6.20%–24.86%), whereas its relative abundance in Yongding was only about 0.45%.

#### 3.3.2 Analysis of microbial diversity

Venn diagrams illustrating the shared and unique microbial species among cigar tobacco leaf samples from the five production areas in Hunan are presented in Figure 4. A total of 121 bacterial and 79 fungal species were common to all five regions. Samples from Cili County had the highest numbers of unique bacterial and fungal species, suggesting that regional environmental conditions substantially promote the enrichment of specific microorganisms.

**FIGURE 4:**
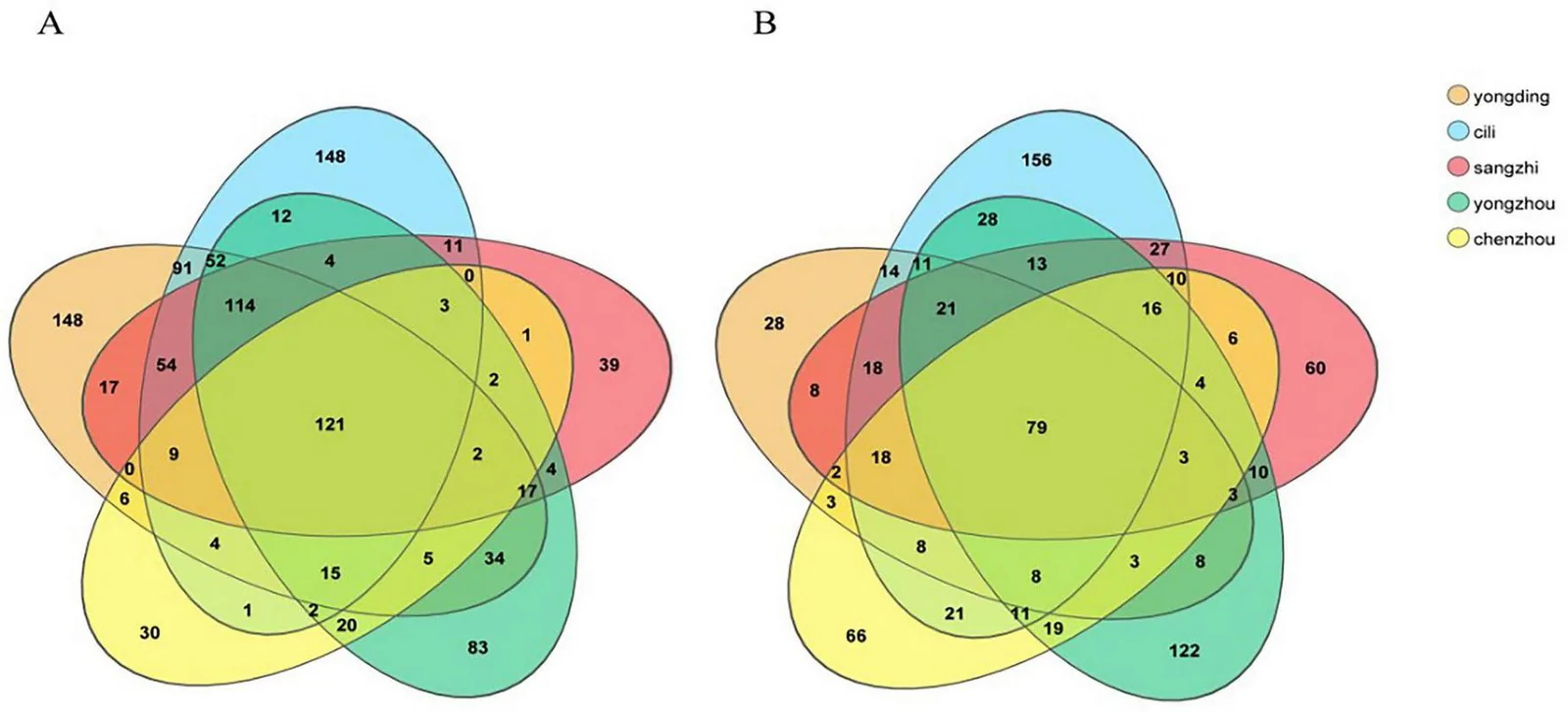
Numbers of unique and shared bacterial **(A)** and fungal **(B)** microbiota in cigar tobacco leaves from different areas. Venn diagrams showing the overlap of bacterial **(A)** and fungal **(B)** species among the five production areas (Yongding, Cili, Sangzhi, Yongzhou, and Chenzhou). The numbers in each region represent the number of species unique to that area, while the overlapping regions indicate species shared among areas. A total of 121 bacterial and 79 fungal species were shared across all five production areas. Samples from Cili County harbored the highest numbers of unique bacterial and fungal species.

ANOSIM based on the combined bacterial and fungal community data across the five production areas (Figure 5A) yielded R = 0.50 and *P* = 0.001, indicating that inter-group differences were significantly greater than intra-group differences and suggesting that geographical origin is the primary factor shaping the overall microbial community differentiation.

**FIGURE 5:**
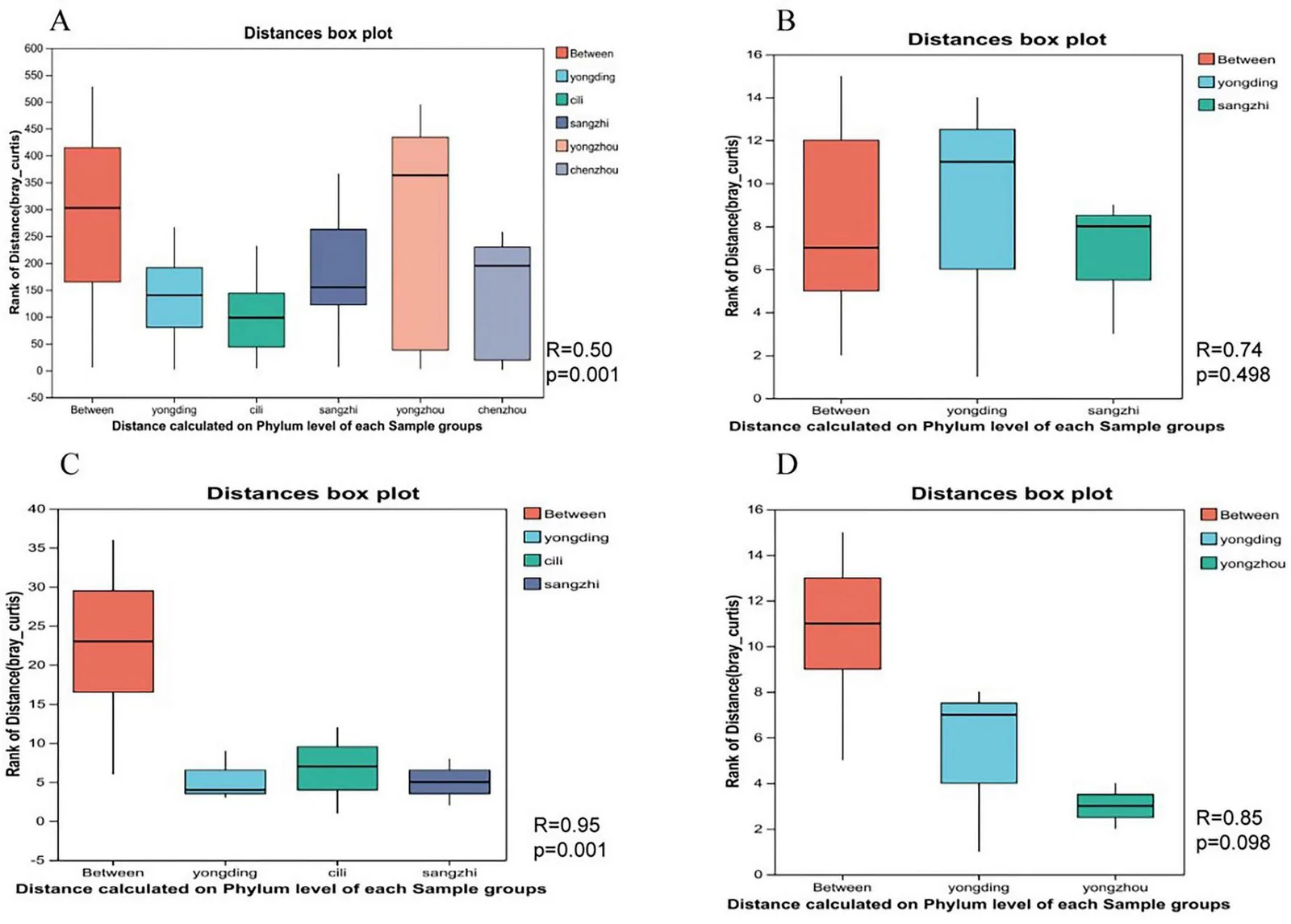
Analysis of microbial community differences among different producing areas. **(A)** Overall comparison of the five production areas. **(B)** Comparison among production areas for Yunxue No. 2. **(C)** Comparison among production areas for Cuba No. 7. **(D)** Comparison among production areas for Habanos. The y-axis represents the rank of distance. R and *P*-values are indicated in each panel. The results indicate that geographical origin was the primary factor driving microbial community differentiation [**(A)** R = 0.50, *P* = 0.001)] with the same variety (Cuba No. 7) showing highly significant inter-regional differences [**(C)** R = 0.95, *P* = 0.001].

For the same variety grown in different regions, Cuba No. 7 (Figure 5C) showed R = 0.95 and *P* = 0.001, reflecting highly significant regional differences. In contrast, Habanos and Yunxue No. 2 (Figures 5B,D) did not show significant regional variation, implying that the effect of variety on microbial selection depends on the specific cultivar.

When comparing different varieties within the same production area (Figure 6), the community difference between Cuba No. 7 (TL-2) and Habanos in Yongding District (TL-3) was small, whereas the difference between these two and Yunxue No. 2 (TL-1) was significant. In Chenzhou, Yongzhou, Sangzhi County, and Cili County, different varieties within each region exhibited differences, but none reached statistical significance. These findings further confirm that the influence of production area environment on microbial communities is stronger than that of variety.

**FIGURE 6:**
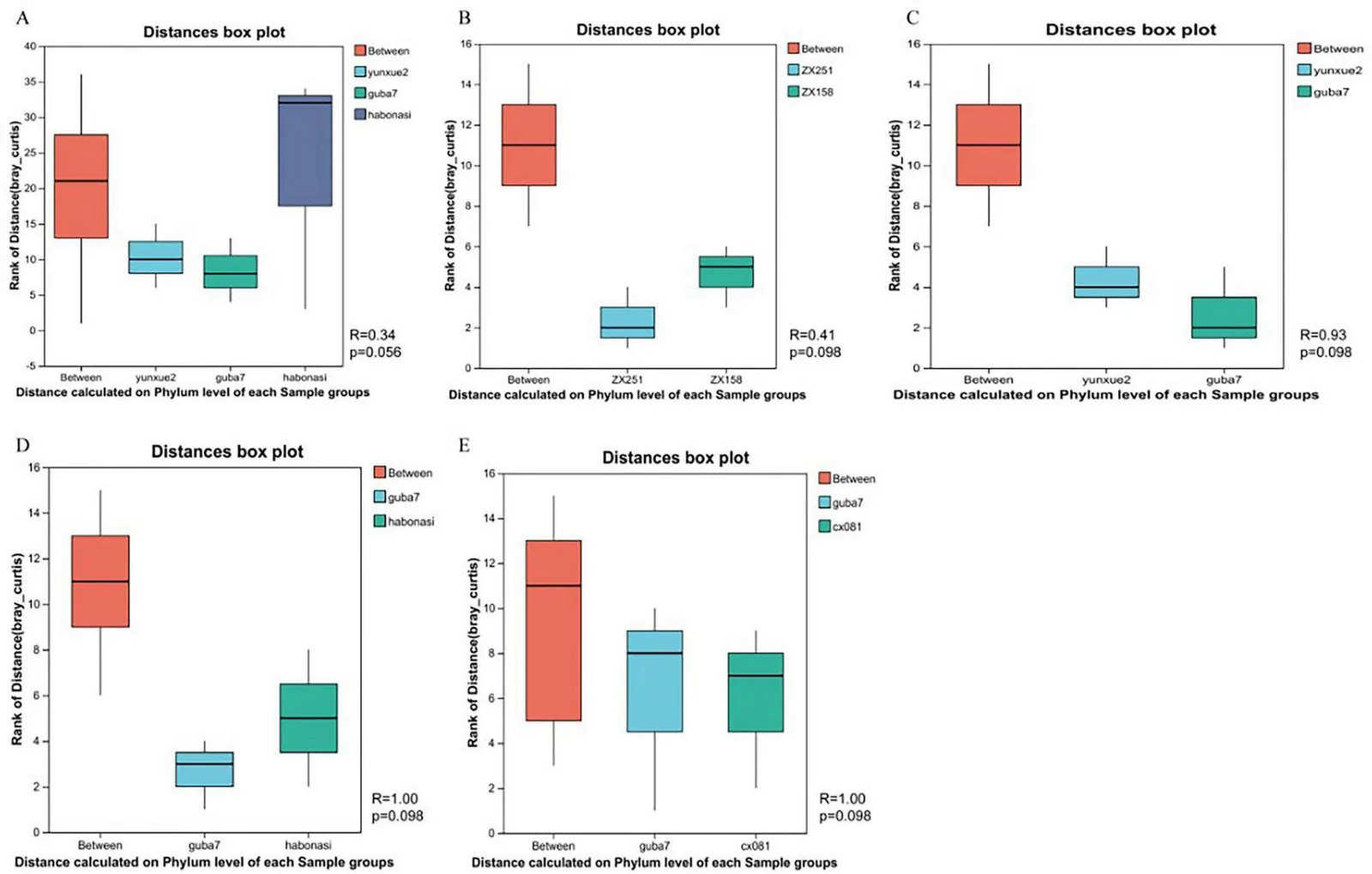
Analysis of microbial community differences among varieties in different areas. **(A)** Analysis of differences among various varieties in Yongding District. **(B)** Analysis of differences among various varieties in Cili County. **(C)** Analysis of differences among various varieties in Sangzhi County. **(D)** Analysis of differences among various varieties in Yongzhou City. **(E)** Analysis of differences among various varieties in Chenzhou City.

### 3.4 Petroleum ether extract content

Petroleum ether extract (PEE) is a complex mixture obtained by extracting cigar tobacco leaves with petroleum ether, comprising volatile oils, resins, fats, fatty acids, waxes, lipoids, pigments, and other lipophilic components. The PEE content in tobacco leaves is closely associated with the overall aroma yield and is considered an important quality indicator. During leaf aging, the extract undergoes transformation and degradation to generate various aroma-active compounds, which further supports its use as a key parameter for evaluating tobacco quality and flavor potential. The PEE contents of the 11 samples are summarized in Table 6 and Supplementary Table 1, ranging from 2.13% to 7.42% overall. Among them, TL-4 exhibited the highest value, while TL-10 and TL-11 had relatively low contents of 2.15% and 2.13%, respectively, indicating substantial variation in total lipid content across the samples.

**TABLE 6:** Petroleum ether extract content of cigar tobacco leaves.

Sample	Petroleum ether extract content (%)
TL-1	5.80 ± 0.26
TL-2	5.13 ± 0.11
TL-3	5.72 ± 0.15
TL-4	7.42 ± 0.23
TL-5	4.82 ± 0.20
TL-6	3.57 ± 0.29
TL-7	6.42 ± 0.25
TL-8	5.33 ± 0.33
TL-9	2.90 ± 0.13
TL-10	2.15 ± 0.26
TL-11	2.13 ± 0.10

### 3.5 Non-volatile organic acid content

Table 7 presents the contents of 14 non-volatile organic acids in the 11 samples. Collectively, oxalic acid, malic acid, and citric acid were the three most abundant organic acids. Malic acid predominated in most samples but exhibited considerable variation (758.19–3261.69 μg/g), with TL-3 and TL-6 showing notably higher contents and TL-10 and TL-11 having substantially lower levels. Citric acid was particularly abundant in TL-7 and TL-11, reaching 2980.95 and 2811.14 μg/g, respectively. Succinic acid was markedly enriched in TL-9, TL-10, and TL-11, with values substantially higher than those in the other samples. Saturated fatty acids (ranging from myristic acid to arachidic acid) were generally present at low concentrations; however, linoleic acid and the combined oleic acid + linolenic acid fraction were relatively elevated in TL-1 and TL-2, whereas they decreased notably in TL-9 and TL-11. Vanillic acid, a phenolic acid, remained relatively stable across samples, with concentrations ranging from 35.73 to 52.89 μg/g. Overall, the organic acid profiles differed substantially among the samples, with the most pronounced disparities observed in the distribution of malic acid, citric acid, succinic acid, and unsaturated fatty acids.

**TABLE 7:** Non-volatile organic acid content in cigar tobacco leaves.

Non-volatile organic acids (μg/g)	Lactic acid	Oxalic acid	Malonic acid	Levulinic acid	Succinic acid	Malic acid	Citric acid	Vanillic acid	Myristic acid	Palmitic acid	Linoleic acid	Oleic acid + linolenic acid	Stearic acid	Arachidic acid
TL-1	7.53 ± 0.57	2147.64 ± 22.93	241.65 ± 7.01	278.45 ± 7.50	37.80 ± 1.48	2447.74 ± 750.80	757.06 ± 30.72	44.65 ± 2.10	20.91 ± 1.35	102.92 ± 5.32	86.40 ± 3.38	136.04 ± 4.86	20.88 ± 0.36	5.20 ± 0.13
TL-2	7.19 ± 0.20	1838.74 ± 61.63	211.48 ± 5.46	48.55 ± 0.97	34.00 ± 0.51	1589.88 ± 531.06	1523.81 ± 75.16	48.65 ± 0.39	14.42 ± 1.20	85.27 ± 2.79	71.96 ± 9.01	117.33 ± 8.97	19.15 ± 3.39	5.38 ± 0.94
TL-3	7.30 ± 0.49	1713.75 ± 29.25	229.56 ± 3.65	20.78 ± 0.39	57.67 ± 0.41	3261.69 ± 614.43	1342.70 ± 32.50	38.15 ± 2.38	13.11 ± 1.68	62.23 ± 1.55	49.86 ± 2.91	87.80 ± 5.42	14.91 ± 1.41	4.15 ± 0.32
TL-4	5.14 ± 3.70	1847.65 ± 139.34	211.34 ± 12.22	29.06 ± 1.40	47.06 ± 2.20	1876.13 ± 490.49	1548.87 ± 163.89	52.89 ± 6.62	14.76 ± 2.78	69.99 ± 3.11	47.45 ± 2.99	89.57 ± 3.28	15.96 ± 0.32	4.85 ± 0.43
TL-5	8.09 ± 0.93	2187.78 ± 60.16	226.84 ± 3.66	95.55 ± 1.74	88.66 ± 1.05	2479.69 ± 626.64	1665.87 ± 94.03	41.63 ± 1.26	11.42 ± 2.64	73.65 ± 0.73	43.43 ± 3.86	92.25 ± 5.50	20.33 ± 3.33	5.92 ± 0.37
TL-6	7.80 ± 0.02	2297.98 ± 75.82	189.35 ± 3.75	14.95 ± 0.55	51.39 ± 1.06	2502.29 ± 684.23	1522.57 ± 8.82	48.56 ± 2.90	9.91 ± 1.54	64.77 ± 2.58	49.00 ± 2.64	96.03 ± 4.16	20.71 ± 0.51	5.21 ± 0.26
TL-7	8.44 ± 0.17	2379.41 ± 58.85	202.28 ± 4.73	23.85 ± 0.39	76.51 ± 1.69	1333.44 ± 497.38	2980.95 ± 52.08	36.16 ± 2.82	10.23 ± 0.28	65.74 ± 1.10	40.30 ± 1.50	96.28 ± 2.59	18.16 ± 0.90	5.33 ± 0.27
TL-8	14.35 ± 13.65	1689.11 ± 98.62	208.60 ± 8.69	29.11 ± 1.25	73.64 ± 3.36	1569.71 ± 385.53	1137.00 ± 24.00	35.73 ± 2.47	10.40 ± 2.55	61.55 ± 0.56	40.53 ± 3.37	76.26 ± 2.68	18.64 ± 1.16	6.00 ± 0.72
TL-9	7.55 ± 0.70	2282.27 ± 73.41	257.71 ± 6.96	16.24 ± 0.23	188.98 ± 2.76	1126.84 ± 406.30	1585.24 ± 20.77	51.33 ± 1.62	10.57 ± 1.69	58.47 ± 0.50	36.63 ± 0.35	64.01 ± 1.34	16.34 ± 0.16	5.91 ± 0.56
TL-10	7.25 ± 0.51	3020.05 ± 139.24	230.23 ± 8.63	13.59 ± 0.41	303.99 ± 6.19	758.19 ± 307.94	1748.20 ± 18.73	49.69 ± 0.48	12.72 ± 3.27	73.97 ± 1.37	23.34 ± 1.12	48.43 ± 2.76	20.55 ± 0.40	6.47 ± 0.76
TL-11	7.12 ± 0.05	2605.24 ± 21.38	188.99 ± 1.27	15.99 ± 0.13	174.84 ± 0.92	824.07 ± 320.56	2811.14 ± 83.52	47.36 ± 1.05	6.02 ± 0.37	68.08 ± 0.98	29.54 ± 2.34	60.56 ± 4.94	22.71 ± 1.10	8.04 ± 0.38

### 3.6 Correlation analysis among microbial composition, chemical components, and sensory indicators

In this study, multivariate statistical methods were applied to systematically explore the associations among microbial communities, chemical components, and sensory attributes. Given the limited sample size, we primarily employed Spearman correlation analysis combined with network analysis to identify microbial genera that were significantly correlated with both chemical constituents and sensory indices, with the aim of revealing potential interaction pathways. Spearman correlation coefficients were calculated pairwise among all microbial genera (based on relative abundance), chemical components, and sensory indicators. Correlations with |R| ≥ 0.6 and *P* < 0.05 were considered statistically significant and retained for constructing the correlation heatmap.

As shown in Figure 7, the positively functioning microbial groups (comprising *Curtobacterium*, *Aureimonas*, *Bacillus*, *norank*_*f*__*Mitochondria*, and *Nigrospora*) collectively enhanced overall aroma, maturity, irritation, and smoothness. This enhancement was achieved by elevating the contents of petroleum ether extract, unsaturated fatty acids, malic acid, and levulinic acid, while reducing succinic acid and oxalic acid. In contrast, the negatively functioning groups (including *Trichoderma*, *Trichomonascus*, unclassified_f__*Nectriaceae*, *Aspergillus*, and *Staphylococcus*) markedly diminished aroma, maturity, and mouthfeel by promoting the accumulation of succinic acid and oxalic acid and depleting beneficial metabolites. Notably, the chemical indicators particularly unsaturated fatty acids, succinic acid, and malic acid, serve as a central regulatory nexus that bridges the microbial community and sensory attributes, offering clear and tractable targets for quality-oriented improvement.

**FIGURE 7:**
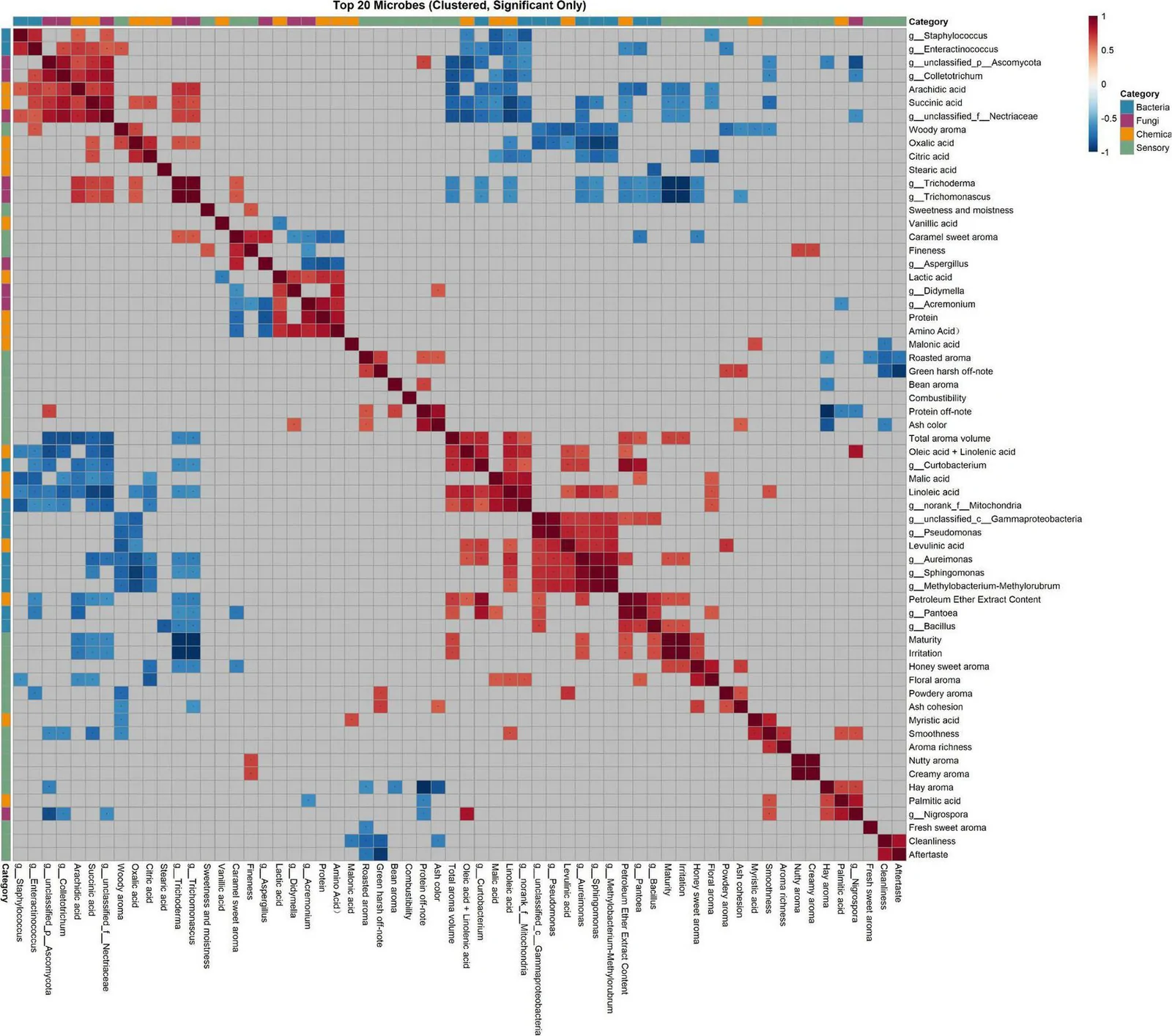
Correlation heatmap of microorganisms-chemical components-sensory indicators.

## 4 Discussion

In this study, we integrated multidimensional datasets—encompassing microbial community composition, chemical profiles, and sensory evaluations—to systematically characterize the microbial community architecture of domestically produced cigar tobacco leaves and its regulatory role in quality development. High-throughput sequencing revealed that the bacterial communities were dominated by *Proteobacteria*, *Cyanobacteria*, *Firmicutes*, and *Actinobacteria*, whereas the fungal communities were overwhelmingly dominated by *Ascomycota*, with relative abundances exceeding 93%. This core compositional pattern is partially consistent with earlier findings (Ye et al., 2021; Zhang et al., 2018); however, discrepancies at the phylum level may be attributed to region- and variety-specific factors (Yan et al., 2022). At the genus level, the predominant bacterial taxa included *Pantoea*, *Pseudomonas*, *Klebsiella*, *Brachybacterium*, and *Curtobacterium*, while the dominant fungal genera comprised *Aspergillus*, *Gibberella*, and *Alternaria*. Collectively, these genera represent key functional groups that underpin the regulation of subsequent flavor metabolism (Reid et al., 1937).

Origin-driven community differentiation emerged as one of the most notable findings of this study. ANOSIM analysis (R = 0.50, *P* = 0.001) confirmed that geographical origin was the dominant factor, as exemplified by the highly significant differences observed for the same variety, Cuba No. 7, across different production areas (R = 0.95, *P* = 0.001). At the bacterial level, *Pseudomonas* dominated in Cuba No. 7 samples from Yongding District and Cili County (Zhangjiajie), with relative abundances of 9.43%–12.67%, whereas its abundance in Sangzhi County was only 0.34%. At the fungal level, *Gibberella* accounted for only 0.45% in Yongding District, but reached 6.20%–24.86% in Sangzhi and Cili counties.

These pronounced origin-driven patterns can be mechanistically explained by a hierarchical dual-filtering assembly model recently established for tobacco-associated microbiomes. In this model, geographical environmental factors predominantly structure soil microbial communities through divergent edaphic properties, while host filtering acts as a universal dominant driver shaping endophytic microbiome assembly (Zhang H. et al., 2026). The same framework has been shown to produce a deterministic soil–plant continuum stratification of bacterial communities, with progressive community simplification and dominance of *Proteobacteria* from bulk soil to aboveground tissues. Our ANOSIM results are consistent with this framework: the strong differentiation of Cuba No. 7 across production areas reflects the combined effects of environment-driven source pool variation and deterministic host selection. This interpretation is further supported by evidence that host phylogeny and functional traits primarily drive phyllosphere microbiome assembly, with plant filtering effects significantly influencing microbial composition and plant-fungal networks exhibiting greater host specificity than plant-bacterial networks (Zhu et al., 2025). Additionally, the strength of host microbiome filtering depends on the proximity to surrounding vegetation sources, and the influence of soil on phyllosphere assembly diminishes when local plant sources are closer or more abundant (Meyer and Lindow, 2026). For cigar tobacco leaves specifically, the microbial community primarily originates from the soil and surrounding environment during field growth, as well as from the air during the drying process (Liu et al., 2025). The processing environment (e.g., drying rooms and fermentation facilities) constitutes a secondary source, and indigenous microorganisms adapted to local temperature, humidity, and tobacco substrate characteristics can rapidly colonize and multiply on the leaves, thereby influencing the composition and structure of the microbial community during subsequent fermentation. Furthermore, microbial species interactions and plant–soil feedback processes can cause historical contingency effects in community assembly, and neighboring plants can rewire these feedback patterns by reshaping root microbiomes. In fruit crops, flavonoid-mediated crosstalk between host plants and rhizosphere microbes has been shown to modulate fruit quality through recruitment of specific beneficial taxa and microbial metabolic reprogramming. Collectively, these findings support the view that origin-driven community differentiation arises from the interplay of environmental selection, host filtering, and microbial dispersal limitation, rather than from a single deterministic force.

The correspondence between community functional traits and quality regulation further elucidated the microbe-driven metabolic mechanisms underlying quality formation. The roles of *Bacillus*, *Pantoea*, *Aureimonas*, and the *Pseudomonas*–*Aspergillus* consortium in promoting tobacco fermentation quality are well-documented. *Bacillus* was dominant and enriched during flue-cured tobacco aging, improving aroma via aromatic amino acid metabolism (Zhu et al., 2025). Co-fermentation with *B*. *velezensis* A2 and *B*. *endophyticus* A4 increased aroma-active metabolites such as butyl butyrate, p-tolyl acetate, and p-cresol (Zhang et al., 2025b), while *B. licheniformis* reshaped surface communities and *B. altitudinis* promoted nitrogenous compound degradation (Zhang J. et al., 2026). *B*. *cereus* A15 activated TCA cycle and terpenoid biosynthesis, enriching guaiacol, damascone, and β-ionone (Liang et al., 2024). In our study, *Bacillus* correlated positively with petroleum ether extract, maturity, and irritation, consistent with these reports. *Pantoea* replaced *Saccharomyces* during low-grade tobacco fermentation and correlated with sensory changes (Shu et al., 2026); its abundance increased during air-curing and correlated with phenylacetaldehyde and acetophenone (Zhang et al., 2025a), and *P*. *ananatis* produces jasmine-like aroma via indole (Zhao et al., 2026). *Aureimonas* is ecologically significant in tobacco-associated communities (Wu et al., 2023; Bai et al., 2026; Zhang Q. Y. et al., 2020), and we found it positively correlated with petroleum ether extract, unsaturated fatty acids, and levulinic acid. *Pseudomonas* and *Aspergillus* synergistically facilitate nicotine degradation and volatile formation (Zhang Q. et al., 2020), and are core drivers during curing and fermentation (Yue et al., 2026).

*Trichoderma* showed dual roles: pre-harvest biocontrol improved upper leaf quality (Su et al., 2025), but in our study it was negatively correlated with maturity and irritation, possibly due to precursor depletion or undesirable metabolites. *Trichomonascus* is associated with mildew (Wei et al., 2024) and influences species evenness (Chen et al., 2024); it correlated negatively with maturity and irritation in our study, indicating suboptimal fermentation. *Staphylococcus* enhanced aroma and reduced irritation in some systems (Pei et al., 2025) and was dominant in rice slurry fermentation (Ren et al., 2024), but in our study it correlated negatively with malic acid, unsaturated fatty acids, and floral notes. This context-dependency highlights species-level functional differentiation.

Collectively, these findings reveal a functional dichotomy: *Bacillus*, *Pantoea*, *Aureimonas*, and *Pseudomonas*–*Aspergillus* promote quality, whereas *Trichoderma* and *Trichomonascus* are associated with deterioration, and *Staphylococcus* exhibits context-dependent effects. This supports the hypothesis that origin-driven microbial differentiation shapes quality through differential metabolic activities of functionally distinct groups. In this study, unsaturated fatty acids, succinic acid, and malic acid emerged as the core metabolic hubs linking microbial communities to sensory quality. Previous research has shown that the levels of nitrogen, sugars, and organic acids in tobacco leaves are associated with the sweetness and mellowness of cigar products (Hu et al., 2022). Petroleum ether extract is widely recognized as a key chemical indicator for evaluating tobacco quality; foliar application of bacterial suspensions has been reported to increase its content, thereby contributing to quality improvement. Moreover, microbial fermentation has been shown to enhance multiple sensory attributes of heat-not-burn tobacco products, including aroma richness, moistness, strength, and reduced irritation (Gao et al., 2010). Collectively, these earlier findings corroborate our present observations that petroleum ether extract and unsaturated fatty acids are positively correlated with overall aroma intensity and maturity.

In summary, this study combined the community ecological patterns revealed by high-throughput sequencing with the metabolite-sensory association network, demonstrating that origin-dominated microbial community differentiation drives the directed accumulation of chemical components through the specific metabolic activities of functional microbial groups, ultimately shaping the sensory quality differences among samples. The functional traits of core dominant genera provide the material basis for flavor regulation, while origin-specific microbial taxa serve as “microbial fingerprints” that contribute differentially to quality formation. This finding not only validates the hypothesis that microorganisms are the core driving force of quality formation, but also provides a theoretical basis for targeted cultivation and fermentation process optimization based on regional microecological regulation: microbial inoculants can be developed targeting positively regulating groups such as *Aureimonas* enriched in Cili County, while temperature and humidity control in Sangzhi County can suppress the over-proliferation of negatively regulating groups like *Trichoderma*, thereby achieving precise quality improvement.

## 5 Conclusion

Microorganisms in cigar tobacco leaves play an important role in shaping the characteristic flavor profiles, with diverse taxa exerting distinct metabolic functions. The geographical origin of the leaves drove pronounced differentiation in microbial community composition, which was associated with the targeted accumulation of key chemical components through the differential metabolic activities of positively and negatively associated functional groups, ultimately contributing to differences in sensory quality and appearance traits. This study provides evidence that microorganisms are important drivers of quality formation and reveals a potential regulatory framework linking origin, microbiota, metabolites, and sensory attributes. These findings offer a scientific basis for further functional validation and for precise quality improvement of cigar tobacco leaves through microecological interventions, such as the development of targeted microbial inoculants and the modulation of environmental factors.

## Data Availability

The original contributions presented in the study are publicly available. This data can be found here: http://www.ncbi.nlm.nih.gov/bioproject/1542265.

## Footnotes

1
https://cloud.majorbio.com
